# The Manifestation of Incidental Findings in Different Experimental Visual Search Paradigms

**DOI:** 10.11621/pir.2022.0409

**Published:** 2022-12-30

**Authors:** Olga S. Rubtsova, Elena S. Gorbunova

**Affiliations:** a HSE University, Laboratory for Cognitive Psychology of Digital Interfaces Users; b HSE University, School of Psychology

**Keywords:** Incidental findings, visual search, subsequent search misses, prevalence effect, target similarity, visual attention, visual perception

## Abstract

**Background:**

Incidental findings are items of visual search that are potentially of significance, but were not the main object of the initial search. They have been previously widely discussed in the field of radiology. However, the underlying perceptual mechanisms of such phenomenon are still unclear.

**Objective:**

The current study aims to examine incidental findings in different paradigms of visual search in order to reveal their primary perceptual aspects.

**Design:**

Two behavioral visual search experiments were conducted. The mixed hybrid search task model was used in the first experiment, while the subsequent search miss effect was employed in the second experiment. The task was to find targets among distractors, according to given instructions. Stimuli material consisted of images of real-life objects that were randomly distributed across the screen for each trial.

**Results:**

Accuracy and reaction time of the participants were analyzed in both experiments. Similar effects were observed for both parameters. Specific targets in the first experiment and typical targets in the second experiment were found significantly faster and more accurately in comparison to categorical and atypical targets. Moreover, this tendency did not depend on the order of target identification. Hence, the prevalence of the targets was revealed to be the primary factor in the case of incidental findings.

**Conclusion:**

The study revealed the emergence of incidental findings in both experiments. Typical or specific targets were detected significantly more accurately, compared to atypical or categorical targets. Subsequent search misses were not detected, suggesting that target prevalence could be a crucial factor that is specific for incidental findings.

## Introduction

Incidental findings are items that were not the primary targets of the visual search, but nonetheless have potential value for the searcher. Initially, they were widely studied in radiology as medical artifacts, unrelated to the main diagnosis (e.g., Berbaum et al., 1990; Beigelman-Aubry, Hill, & Grenier, 2007). Finding signs of cancer while examining a patient with pneumonia might serve as an example of such findings. However, recent work by Wolfe et al. (2017) examined the underlying mechanisms of such phenomena in visual search. The authors used a model specifically designed to compare categorical and specific searches in different conditions. They suggested that incidental findings were associated with categorical searches, while typical targets corresponded to specific searches. Specific search is a search for targets with a specific identity (for example, when one searches for their own keys in a bag), and categorical search (Yang & Zelinsky, 2009) is a search for all targets from one category (for example, looking for vegetables in the store). Specific search is simpler since it is based only on one representation of the primary target. Categorical search, however, demands more attentional resources, since there is no clear representation of targets. Experimental research supports these assumptions. A good illustration is a study by Maxfield and Zelinsky, which investigated the influence of categorical hierarchy on visual search (Maxfield & Zelinsky, 2012). Within the study, searchers were primed with subordinate (e.g., dalmatian), basic (e.g., dog) or superordinate (e.g., animal) category names, which helped to guide and clarify searches. It was revealed that the guidance increased with increasing specificity of the category labels. Hence, targets classified by a subordinate, or narrower, category, were easier to find. In a similar manner, it can be harder to find less defined targets in the case of the incidental findings phenomenon. The searchers do not have clear representations of such items, although they constitute the general category of medical abnormalities.

Incidental findings seem to be closely connected to the effect of the prevalence of targets. It was shown in several studies that targets of high prevalence are typically identified much faster and more precisely than those of low prevalence (e.g., Hout et al., 2015). In case of incidental findings, targets of low prevalence would correspond to less defined categorical items. The underlying mechanism of the low-prevalence effect is possibly based on forced-choice decisions made by searchers while performing the task. The thresholds responsible for making the decision to continue the search after finding one target can be altered by various factors. Research shows that in the case of low-prevalence targets, the threshold for abandoning further search is significantly lower compared to the high-prevalence condition (Wolfe, Van Wert, 2010).

One of the main issues when it comes to identifying the real mechanisms of incidental findings is the problem of experimental paradigms used for the investigation of the studied phenomena. It is a common practice for researchers to choose a standard paradigm, which enables the detection of a studied effect and which has already shown its effectiveness in previous experimental projects. However, for novel effects of visual search, such as incidental findings, the question arises of whether to choose one of the existing paradigms of visual search or to develop a new one. There are experimental models used in visual search research that seem to be suitable for studying incidental findings. Potentially, different paradigms could aid the study of various aspects of this phenomenon, since they have not yet been precisely defined in terms of the underlying cognitive mechanisms. One model for research, closely linked to the hybrid search paradigm, was used by Wolfe and co-workers (Wolfe et al., 2017). The distinctive characteristic of the hybrid search paradigm is that it involves visual search from memory (e.g., Schneider & Shiffrin, 1977; Wolfe, 2012). This is advantageous, since it resembles a visual search in real-life conditions. Another possible method, optimal for incidental findings, could be a subsequent search misses (SSM) paradigm. SSM are the effect of a significant decline in the accuracy of the identification of the second target (Adamo, Cain, & Mitroff , 2013; Adamo et al., 2019; Fleck et al., 2010). Originally, SSM were referred to as “satisfaction of search” and were widely studied in radiology (e.g., Tuddenham, 1962; Berbaum et al., 1994). SSM are related to primary targets of search, as opposed to incidental findings, and the target found second is typically very similar to the target found first. Nevertheless, SSM resemble incidental findings as perceptual phenomena of visual search. Both effects can be related to the identification of additional targets following the detection of the first target. Therefore, the visual search errors related to them might be due to perceptual biases or resource limitations related to the processing of the first target. This is specifically important, since in experimental conditions both incidental findings and SSM are studied within multiple target search paradigms. Hence, it is crucial to understand how to behaviorally dissociate between the two phenomena. There were several studies that revealed the factors responsible for the accuracy shift in the case of the detection of the second target. Some studies illustrated that perceptually similar targets were identified more accurately (e.g., Gorbunova, 2017), while others showed the role of their categorical identity (Biggs et al., 2015) as more significant. All aspects considered, the similarity of targets may play a crucial role in the emergence of the discussed visual search effects.

Different experimental paradigms allow the identification of various factors that lead to the emergence of specific perceptual effects. The traditional SSM paradigm provides very high target-distractor similarity. When targets closely resemble distractors, the overall visual search task becomes much harder (Duncan & Humphreys, 1989). Therefore, the SSM errors might be due to the perceptual noise created by the distractors. On the other hand, the mixed hybrid search model includes objects from different categories, therefore creating a much larger perceptual variance among all items on display. Hence, target-distractor similarity may be a factor that behaviorally separates incidental findings from SSM. However, the two paradigms also differ in terms of target prevalence representation. Wolfe and colleagues’ model was created to easily manipulate the percentage of particular targets on screen. In standard SSM paradigms, this parameter is not varied. As such, a bias towards specific targets throughout the task is not created. Rather, the emphasis is put on the bias created by the initially identified target in each individual experimental trial. This difference might be crucial in differentiating incidental findings from SSM. If target prevalence is manipulated in both experimental paradigms, the results could specify the perceptual underlying mechanisms of these phenomena.

The objective of this research was to study incidental findings using two different experimental visual search paradigms: a mixed hybrid search model developed by Wolfe and colleagues, and an SSM paradigm. The mixed hybrid model involves searching for several targets from memory, some of which are defined by category, while others are specific. The procedure is separated into several blocks, so that targets and distractors are defined for each individual block separately. In contrast, the SSM paradigm involves searching for initially defined targets during the whole procedure. Target prevalence was chosen to be manipulated in both experimental paradigms in order to reveal its specificity to incidental findings. The main criterion for identifying incidental findings was the absence of statistical differences between conditions with one target (categorical or non-typical) and two targets, as suggested by Wolfe et al. (2017). Therefore, if incidental findings emerge in both experimental models, it suggests that target prevalence is indeed the crucial factor for distinguishing the described perceptual phenomena. However, if SSM were to be found in the paradigm for SSM research, it would mean that target prevalence is not specific for incidental findings, and there are likely other perceptual factors that play a significant role.

## Experiment 1

### Methods

#### Participants

There were originally 17 participants in this experiment. The sample size was based on the experimental work by Wolfe et al. (2017), who originally introduced the mixed hybrid search model. Slightly more participants were invited, in order to compensate for distant data collection. All were required to have normal or corrected to normal vision and to have no neurological or psychological problems. Every participant read and signed the informed consent. Data from 3 participants were excluded from further analysis, due to misunderstanding of the instructions. Therefore, the final sample consisted of 12 females and 2 males, their ages ranging from 18 to 36 years old (*M* = 24.14, *SD* = 5.14).

#### Stimuli material

Eight categories of food were chosen as stimuli material: vegetables, fruit, groceries, drinks, meat products, dairy products, bakery, and desserts. For each of those categories, ten different objects were chosen as stimuli. The images were taken from open stock-images bases and modified in Adobe Photoshop to isolate the objects from the background and change the image size. The stimuli represented real life objects in order to correspond to the experimental task, so primary perceptual factors like color and brightness were not specifically controlled. However, since different stimuli were randomly distributed across trials, possible systematic biases related to such factors were eliminated. Each image was 160x120 pixels in size, vertically oriented. Stimuli were presented on a plain white background. There were also two additional buttons “NO” and “OK” for reporting the absence of the targets.

Overall, six experiments were created with the following conditions: one specific target (36% of tasks), two specific targets (16% of tasks), one categorical target (9% of tasks), two categorical targets (1% of tasks), both specific and categorical targets in the same task (8% of tasks) and no targets (30% of tasks). The percentage distribution is similar to that in experiments by Wolfe et al. (2017). These conditions were then distributed among three experimental blocks: specific, categorical, and mixed. The blocks differed in the type of search, which was specified in the instructions. Within a specific block particular objects would be searched for, in a categorical block the search would be for all objects from a given category, and a mixed block was a combination of those two types of search. The mixed block was critical in this experiment, since it implied both specific and not clearly defined targets, representing incidental findings.

The stimuli were distributed randomly across the screen (1248x640 pixels) within a 5 by 5 invisible grid. Participants could move along up to 55 pixels horizontally and up to 4 pixels vertically randomly from the centers of the cells in each trial. Overall, there could be 4, 8, or 12 stimuli in each individual trial, the number of targets varied from 0 to 2.

#### Procedure

The experiment was conducted remotely on the participants’ computers. They could use any computer with any monitor, but they were specifically required not to use a smartphone or tablet. The participants were sent all the necessary materials, including video-instructions and the experiment files. Before running the experiment, the participants were asked to look through the list containing all the images of stimuli in order to familiarize themselves with which object belonged to which category. After that they were asked to begin the experiment in quiet, comfortable conditions. They were also required to use a computer mouse and a space bar during the experiment.

When the participants ran the experiment, instructions describing the task appeared. It was stated that the task resembled a “grocery shopping” task, and the participants would need to find objects, based either on their specific labels or the name of the category. The labels appeared before the start of each experimental block. The objective was to remember objects or category names and then search for the targets as quickly as possible. As soon as the target was found, it needed to be clicked on using a computer mouse. The buttons “OK” and “NO” served for reporting the absence of targets in conditions with only one or no targets. After the end of each task, the participants could rest if necessary and begin the new task by pressing the spacebar.

Each specific, categorical, and mixed block was evenly divided into two blocks, making six separate blocks. The participants had a chance to rest in between the blocks and begin a new one by pressing the spacebar. Before each block, four labels of objects or category names appeared for 12 seconds. In the specific block there were four labels of specific objects, in the categorical block there were four names of different categories, while in the mixed blocks there were two specific labels and two category names. The labels and category names for each block were chosen at random. The order of the tasks within each block was random. Following the initial instructions, there was a training block consisting of 20 trial tasks to enable the participants to practice and contact the experimenter if anything was unclear. Next, the main part of the experiment began. This consisted of 820 tasks in total.

### Results

Accuracy and reaction time for both mouse clicks were analyzed. The condition with no targets was excluded from the analysis, since it was used as a control to determine the participants’ attention to a given instruction and did contain any relevant data. Accuracy and reaction time were analyzed for conditions with one specific target, one categorical target, two specific targets, two categorical targets, and the condition with both types of targets present together. Moreover, these conditions were analyzed separately for each experimental block: specific, categorical, and mixed. It was necessary to examine the errors, depending on the type of search.

The error analysis was carried out for different experimental conditions. For experiments with no targets, the accuracy and reaction time were calculated using the times when participants successfully clicked the “NO” button twice. For experiments with one specific or categorical target, the accuracy and reaction time were calculated using times when the click on the target was followed by a click on “OK” button. For experiments with two specific or categorical targets, the accuracy and reaction time were measured for the second target, regardless of the order in which the targets were clicked. For the experiments with both target types (in the mixed block), the accuracy and reaction time were calculated for the categorically defined target, but only if it was found after the specific one. Accuracy and reaction time then were compared for the relevant experimental conditions. Reaction time was analyzed for correct response trials. Reaction times (RTs) greater than M+2SD and less than M-2SD were excluded from further analysis.

IBM SPSS Statistics v. 22.0.0.0 was used for data analysis. In order to determine which type of search (specific or categorical) was more accurate, two-way ANOVA was used. Moreover, multiple paired sample t-tests were applied for pairwise comparisons of different conditions with Bonferroni adjustments. For analyzing the effects within the mixed block repeated measures, ANOVA and pairwise comparisons with Bonferroni-Holm adjustment were used. The Greenhouse-Geisser corrections were applied when Mauchly’s sphericity tests were significant.

Incidental findings were detected based on the accuracy parameter. If there were no significant differences between dual- and single-target tasks, incidental findings would be detected. Otherwise, in the case of the significant decrease in accuracy related to dual-target trials, SSM would be detected. The reaction time parameter was considered secondary to the accuracy parameter. It was used to further clarify the differences between different experimental conditions, particularly between categorical and specific visual search.

#### Accuracy

**Figure 1.**
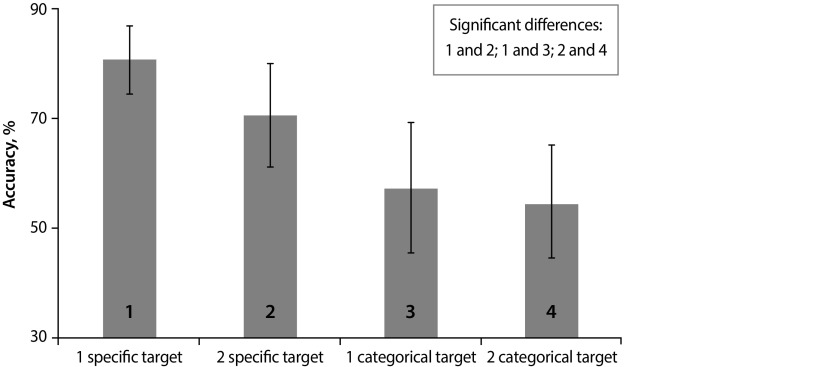
The results of accuracy analysis for conditions in specific and categorical blocks

*Note*. Error bars represent 95% confidence intervals. Statistical differences indicate the results of paired sample t-tests.

Two-way ANOVA revealed a significant effect of the target type factor (*F*(1,13) *=* 30.314, *p* < .001, η^2^_p_= 0.7) and the number of targets factor *F*(1,13) *=* 10.013, *p* = .007, η^_2_^_p_= 0.435). The factor interaction was insignificant (*F*(1,13) *=* 2.083, *p* = .173, η_p_^2^ = 0.138). The search for specific targets was more accurate in conditions with one target (*t*(13) = 6.661, *p* < .001, *d* = 1.31) and two targets (*t*(13) = 3.144, *p* = .016, *d* = 2.91). The participants were significantly more accurate in detecting the only target in a task compared to two targets, but only in the specific block (*t*(13) = 3.267, *p* = .018, *d* = 5.23). The accuracy did not differ significantly depending on the number of targets (*t*(13) = 0.852, *p* = .409, *d* = 0.14). The results are presented in *Figure 1*.

**Figure 2.**
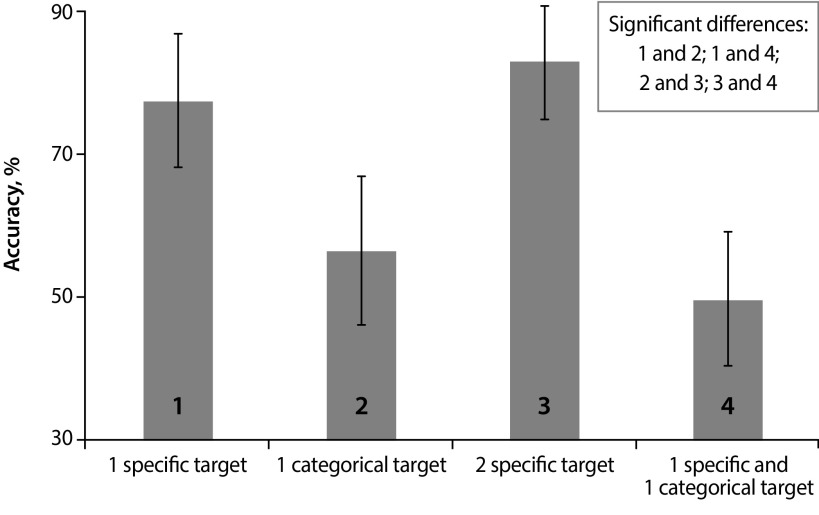
The results of accuracy analysis for the mixed block

*Note*. Error bars represent 95% confidence intervals. Statistical differences indicate the results of pairwise comparisons.

In the mixed block, ANOVA showed a significant effect of the experimental condition factor: *F*(3,39) = 35.012; *p* < .001; η_p_^2^ = 0.729. Pairwise comparisons with Bonferroni adjustments revealed significant differences between the following conditions: one specific target and one categorical target (*p* < .001), one specific target and both specific and categorical targets in the same trial (*p* < .001), one categorical target and two specific targets (*p* = .001), and two specific targets and both specific and categorical targets in the same trial (*p* < .001). The results are presented in *Figure 2*.

#### Reaction time (first click)

**Figure 3.**
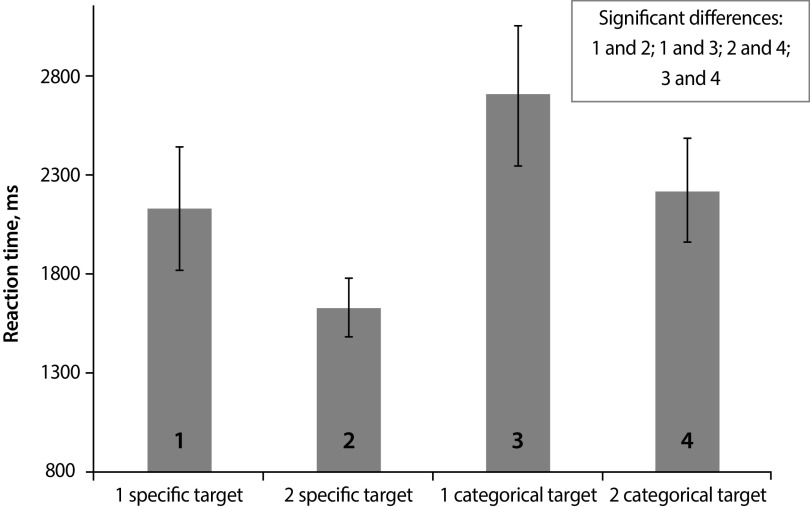
The results of the reaction time (first click) analysis for different experimental conditions in specific and categorical blocks

*Note*. Error bars represent 95% confidence intervals. Statistical differences indicate the results of paired sample t-tests.

Two-way ANOVA revealed a significant effect of the target type factor (*F*(1,13)*=* 48.481, *p* < .001, η^2^_p_= 0.789) and the number of targets factor (*F*(1,13) *=* 28.938, *p* <.001, η^2^_p_= 0.69). The factor interaction was insignificant (*F*(1,13) *=* 0.012, *p* = .916, η^2^_p_= 0.001). It took significantly less time to find a specific target than a categorical one in experiments with either one target (*t*(13) = - 4.459, *p* = .001, *d*=0.87) or two targets (*t*(13) = - 5.758, *p* < .001, *d* =1.42). Furthermore, it took more time to identify a single target as opposed to one of two targets. This was true for both specific (*t*(13) = 4.5, *p* = .002, *d* = 1.06) and categorical blocks (*t*(13) = 3.671, *p* = .003, *d* = 3.13). The results are illustrated in *Figure 3*.

**Figure 4.**
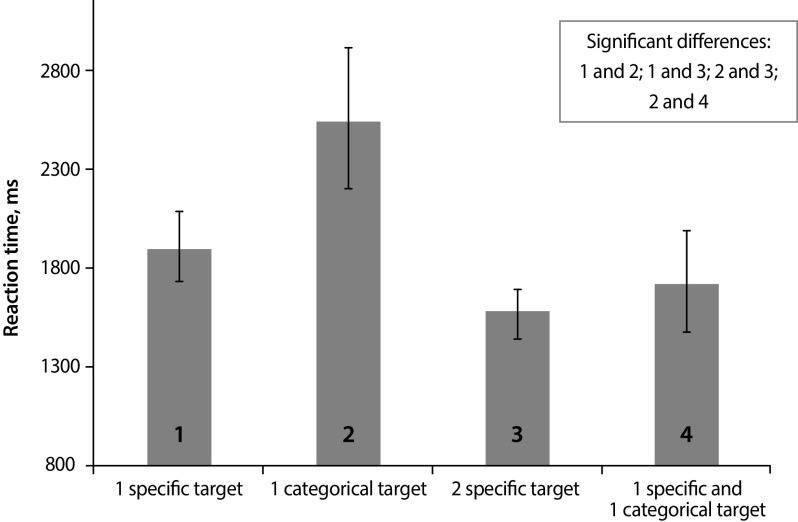
The results of reaction time (first click) analysis for the mixed block

*Note*. Error bars represent 95% confidence intervals. Statistical differences indicate the results of pairwise comparisons.

In the mixed block, ANOVA showed a significant effect of the condition factor: *F*(3,39) = 32.071; *p* < .001; η_p_^2^ = 0.712. Pairwise comparisons with Bonferroni adjustments revealed significant differences between the following experimental conditions: one specific and one categorical targets (*p* = .001), one specific and two specific targets (*p* < .001), one categorical and two specific targets (*p* < .001), and one categorical target and both specific and categorical targets in the same task (*p* = .001). The results are presented in *Figure 4*.

#### Reaction time (second click)

Two-way ANOVA revealed a significant effect of the target type factor (*F*(1,13) *=* 17.556, *p* = .001, η^2^_p_= 0.575) and the number of targets factor (*F*(1,13) *=* 34.542, *p* < .001, η^2^_p_= 0.727). Moreover, the effect of factor interaction detected was significant (*F*(1,13) *=* 26.36, *p* < .001, η_p_^2^ = 0.67). Due to this significant interaction, an additional one-way ANOVA was conducted separately for specific and categorical targets (the factor being the number of targets), and another for one target and two targets conditions (the factor being target type).

**Figure 5.**
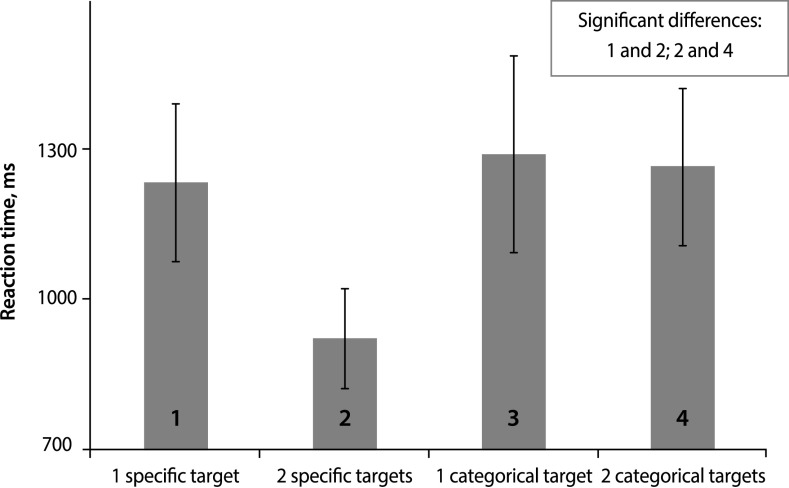
The results of reaction time (second click) analysis for conditions in specific and categorical blocks

*Note*. Error bars represent 95% confidence intervals. Statistical differences indicate the results of paired sample t-tests.

The additional one-way ANOVA revealed the significant effect of the number of targets for specific (*F*(1,13) *=* 45.394, *p* < .001, η^2^_p_= 0.777) but not categorical targets *(F*(1,13)*=* 4159.197, *p* = .469, &^2^ = 0.041). Hence, the participants were significantly quicker to find the second specific target than to report the absence of the second specific target (*p* < .001). However, such a pattern was not found for categorical targets. In this case, it took a statistically similar amount of time to report the second target as it did its absence (*p* = .469). Regarding the effect of target type, it was signififiant for conditions with two targets (*F*(1,13) *=* 35.525, *p* < .001, η^2^ = 0.732) and insignificant for conditions with only one target (*F*(1,13) *=* 1.112, *p* < .311, η^2^ = 0.079). The participants were significantly quicker to click on the second specific target than the categorical one (*p* < .001), but they tended to require an equal amount of time to report the absence of the second target, whether specific or categorical (*p* = .311). The results are illustrated in *Figure 5*.

**Figure 6.**
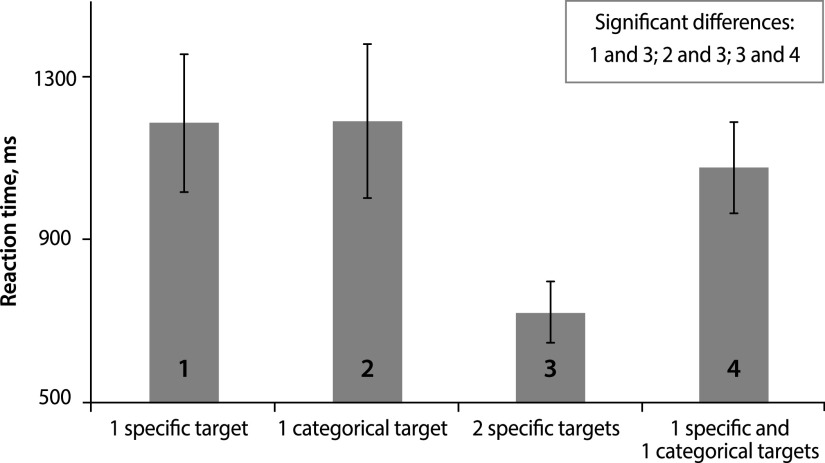
The results of reaction time (second click) analysis for the mixed block

*Note*. Error bars represent 95% confidence intervals. Statistical differences indicate the results of pairwise comparisons.

In the mixed block repeated measures, ANOVA revealed a significant effect of the condition factor: *F*(2,24) = 26.323; *p* < .001; η_p_^2^ = .669. Pairwise comparisons with Bonferroni adjustments revealed significant differences between the following conditions: one specific target and two specific targets (*p* <.001), one categorical target and two specific targets (*p* = .001), and two specific and both specific and categorical targets in the same task (*p* < .001). The results are illustrated in *Figure 6*.

### Discussion

The results of the accuracy analysis within specific and categorical blocks illustrate that the search for categorically defined targets was significantly more prone to errors. The accuracy in detecting specific targets was higher for experiments with both with one and two targets. This is similar to the findings obtained in the research by Wolfe and colleagues, where error rates were significantly higher for categorical targets (Wolfe et al., 2017). The findings are supported by the perceptual set hypothesis, since specific objects are better represented in the working memory, and visual attention is guided towards them (Kristjánsson & Campana, 2010). Categorical targets are less precisely defined, therefore the search for such items is less efficient. Similar results were reported in the study by Maxfield and Zelinsky, where the effects of the category level were studied and it was found that the less defined the target, the lower the accuracy (Maxfield & Zelinsky, 2012). As well as that, there was the effect of SSM for the specific block only: the accuracy declined significantly for the condition with two targets compared with the condition with one target. However, this was not the case for the categorical block: there were no statistically significant differences between the corresponding conditions. This may be explained by the overall difficulty of categorical search, particularly because the participants were accurate in no more than 60% of the trials.

Within the mixed block, there were no significant differences between the experiments with both specific and categorical targets and the experiments with only one categorical target. This finding implies that, by definition, no SSM were observed in this block. It also corresponds to the results of Wolfe and colleagues’ experiment (Wolfe et al., 2017). This is interesting since incidental findings were indeed separated from other visual search phenomena in their study. Furthermore, in the mixed block, as in the other blocks, the search for one specific target was significantly more accurate than the search for one categorical target. Furthermore, the accuracy in the experiments with one categorical target was far lower than the accuracy in experiments with two specific targets. Hence, categorical search seems to be far less precise than specific search.

The results of the first click reaction time analysis further clarify the differences between categorical and specific search. The identification of a specific target was significantly quicker in tasks with both one and two targets in specific and categorical blocks. The same effect was observed in the mixed block experiments with one specific and one categorical target. These findings, once again, resemble those reported in papers by Wolfe (Wolfe et al., 2017) and Maxfield (Maxfield & Zelinsky, 2012). Hence, it can be assumed that finding a categorically defined target takes more time than a specific one. The participants were also significantly quicker to find the first target in tasks with two targets compared to tasks with one target. This suggests that, statistically, it takes less time to find at least one out of two present targets, rather than to find the only present target. This is typical for visual search experiments, as supported by previous experiments and known data (e.g. Kwak, Dagenbach, & Egeth, 1991; Moraglia, 1989).

With regards to the reaction time of the second click for specific and categorical blocks, it took significantly less time to detect the second target if it was specific. Moreover, it took significantly less time to find the second specific target compared to reporting its absence. This was true for both specific and mixed conditions. This finding illustrates the higher probability of detecting the second present target before searching through all present distractors. However, in the categorical block, there were no significant differences between the mentioned conditions. It took a statistically similar amount of time to detect either the second target or report its absence. A possible explanation for this finding is that the time required to make a decision is increased, whether the observed item is a target or not, due to poorly defined target representation. As previously discussed, in the case of specific targets such decisions are made quicker, due to both attentional guidance and distinct perceptual representations of targets. Furthermore, the reaction time for tasks with one target was not significantly different between all three experimental blocks. This is a typical finding since set sizes were evenly distributed among the various experimental conditions, meaning it would take the same amount of time to report the absence of the second target.

## Experiment 2

### Method

#### Participants

There were originally 24 participants in this experiment. The sample size was based on previous experimental research on SSM (e.g., Gorbunova, 2017). All the participants confirmed *via* Google forms (https://www.google.com/forms/about/) that they had normal or corrected to normal vision and did not have any neurological or psychological problems. Data from one participant were excluded from further analysis, due to misunderstanding of the instructions. Therefore, there were 21 females and 2 males. Their ages ranged from 19 to 34 years old (*M* = 22.22, *SD* = 4.13). The participants were given 100 rubles each for participating in the experiment.

#### Stimuli

Food images were used as targets and distractors belonged to several categories: cars, furniture, hats, musical instruments, and shoes. Fruits and vegetables corresponded to typical targets, while spices corresponded to non-typical targets. There were five objects chosen for each category and 60 images in total. The images were taken from open stock-images bases and modified in Adobe Photoshop to isolate the objects from the background and change the image size. Each image was 140x100 pixels in size, vertically oriented. Stimuli were presented on a plain white background. There were also two additional buttons made for participants’ answers, they contained the words “NO” and “OK” correspondingly.

The salience of the two types of targets was varied. Fruits and vegetables were used as typical targets, while spices were non-typical targets. There were five experimental conditions: two typical targets (18% of tasks), one typical target (37% of tasks), no targets (30% of tasks), one non-typical target (5% of tasks), and both typical and non-typical tasks (10% of tasks).

The stimuli were distributed randomly across the screen (1248x640 pixels) within a 5 by 5 invisible grid. There could be 12, 16, or 20 stimuli in each individual trial. The number of targets could be 0, 1, or 2.

#### Procedure

The experiment was conducted online using Pavlovia software (https://pavlovia.org/). The participants used their personal computers and were required not to use smartphones or tablets. They were instructed to search for food among objects from other categories. The participants were informed that they could find 0, 1, or 2 targets in each individual task. They were asked to perform the task as quickly as possible. They used a computer mouse to click on targets and the buttons at the bottom of the screen in order to report the presence or absence of targets, similar to Experiment 1. After the end of each task, the participants could rest if necessary and begin the next task by pressing the spacebar.

The first 60 tasks did not contain non-typical targets, and the order of presentation in the following trials was randomized among all five experimental conditions. There were 495 tasks in the main block of the experiment.

### Results

The analysis was the same as for the mixed block in Experiment 1.

#### Accuracy

**Figure 7.**
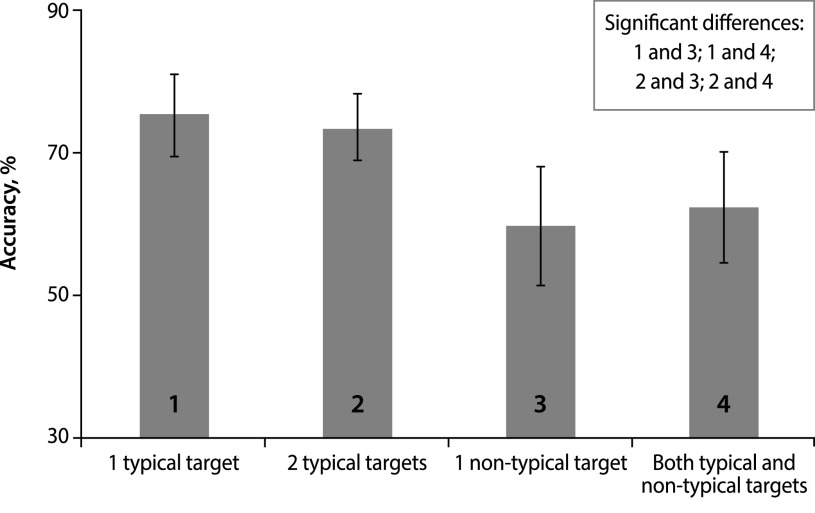
The results of accuracy analysis for Experiment 2

*Note*. Error bars represent 95% confidence intervals. Statistical differences indicate the results of pairwise comparisons.

Repeated measures ANOVA revealed the significant impact of the experimental condition factor: *F*(2,50) = 10.671; *p* < .001; η_p_^2^ = 0.327. Pairwise comparisons with Bonferroni-Holm adjustments revealed significant differences between the following conditions: one typical and one non-typical target (*p* < .001), one typical target and both typical and non- typical targets in the same task (*p* = .015), two typical targets and one non-typical target (*p* = .009), and two typical targets and both typical and non-typical targets in the same task (*p* = .035). The results are presented in *Figure 7*.

#### Reaction time (first click)

**Figure 8.**
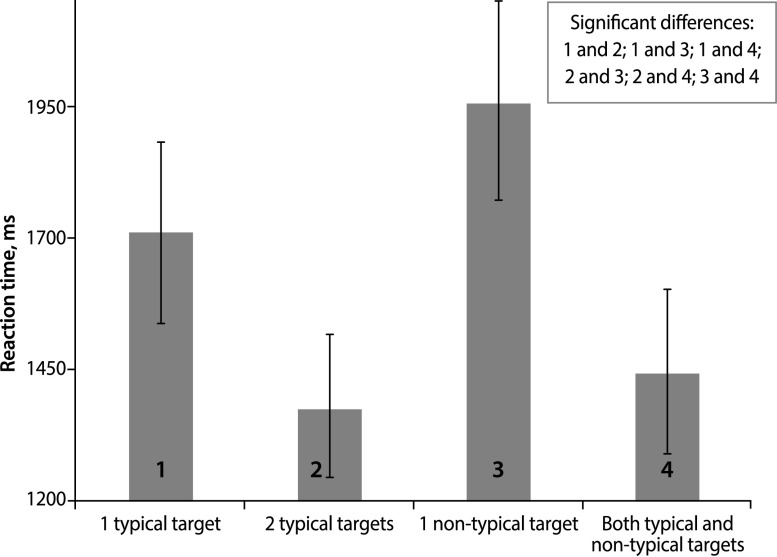
The results of reaction time (first click) analysis for Experiment 2

*Note*. Error bars represent 95% confidence intervals. Statistical differences indicate the results of pairwise comparisons.

Repeated measures ANOVA revealed the significant impact of the experimental condition factor: *F*(2,50) = 144.546; *p* < .001; η_p_^2^ = 0.868. Pairwise comparisons with Bonferroni-Holm adjustments revealed significant differences between the following conditions: one typical target and two typical targets (*p* < .001), one typical target and one non-typical target (*p* < .001), one typical and both typical and non-typical targets in the same task (*p* < .001), two typical and one non-typical target (*p* < .001), two typical and both typical and non-typical targets in the same task (*p* = .015), and one non-typical target and both typical and non-typical targets in the same task (*p* < .001). The results are presented in *Figure 8*.

#### Reaction time (second click)

**Figure 9.**
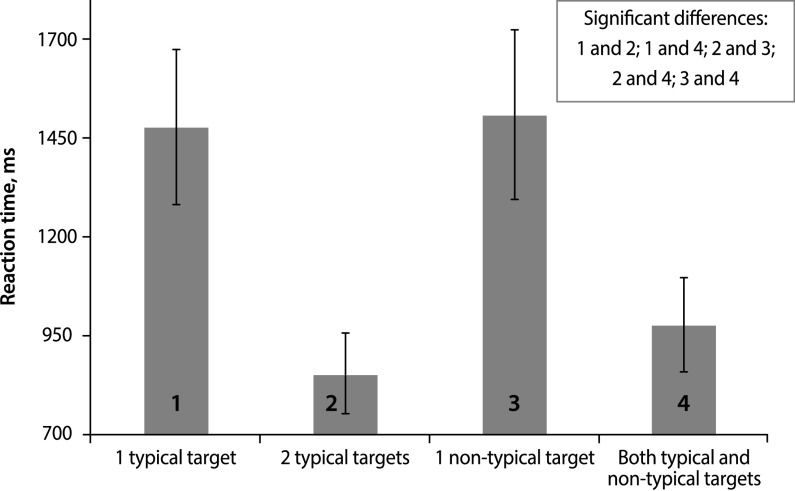
The results of reaction time (second click) analysis for Experiment 2

*Note*. Error bars represent 95% confidence intervals. Statistical differences indicate the results of pairwise comparisons.

Repeated measures ANOVA revealed the significant impact of the condition factor: *F*(1,27) = 47.033; *p* < .001; η_p_^2^ = 0.681. Pairwise comparisons with Bonferroni-Holm adjustments revealed significant differences between the following conditions: one typical target and two typical targets (*p* < .001), one typical and both typical and non-typical targets in the same task (*p* < .001), two typical and one non-typical target (*p* < .001),two typical and both typical and non-typical targets in the same task (*p* < .001), and one non-typical target and both typical and non- typical targets in the same task (*p* < .001). The results are presented in *Figure 9*.

### Discussion

There were no significant differences in accuracy between the baseline experimental condition with one non-typical target and the crucial experimental condition with both typical and non-typical targets in the same task. Therefore, no SSM errors were observed, as in Experiment 1. Interestingly, SSM errors were also not detected for tasks with only typical targets. This can be explained by the effect of high target prevalence (Hout et al., 2015). It is important to note that the targets and distractors belonged to different categories. Therefore, the target-distractor similarity was not large. This could be the major factor leading to the overall reduction of task difficulty, as it is easier to find targets when they are perceptually different from the distractors (Duncan & Humphreys, 1989). In this case, targets were also categorically different, which made the guidance of visual search even easier. As expected, the results for the reaction time showed that it took significantly less time to identify typical targets in comparison to non-typical ones. This further supports the assumption that typicality plays a significant role in the effectiveness of visual search, as typical targets have better representations in working memory. This effect was also observed for non-typical targets that were found after typical ones. In compliance with the categorical perception hypothesis, the search becomes guided by the specific characteristics of the initially found target (e.g., Kristjánsson & Campana, 2010). Finally, the participants were significantly quicker to find the first target in the case of two in-trial present targets, as well as to report the second present target in comparison to reporting its absence. These findings were the same as in Experiment 1.

## General discussion

Two visual search paradigms provided different ways of studying the same phenomenon. Incidental findings are defined as targets that do not relate to the primary search goals but are of potential interest to the searcher. The criterion for distinction between incidental findings and the similar effect of subsequent search misses (SSM) was the difference in accuracy between single- and dual-target tasks. Incidental findings can be identified only in the absence of such statistical differences. The main finding of Experiment 1 was the difference between categorical and specific visual search for targets. Categorically defined targets were easier to miss. As initially suggested by Wolfe and colleagues (Wolfe et. al., 2017), incidental findings are most closely associated with such targets. Targets that have clear representations are typically found first, so less attentional resources are lefy for potentially remaining ones. Significantly, there was no decrease in accuracy for finding the second target after the first one. This distinguishes this effect from previously described SSM. Hence, accuracy in this case does not simply depend on the order in which the targets are identified, but rather on the search characteristics themselves. Similarly, no SSM were detected in Experiment 2, although the standard SSM experimental paradigm was used. Notably, though, the search was categorical in this experiment, and the targets differed in typicality. It was shown that the search for typical targets was significantly more accurate, alike to the search for specific targets in Experiment 1. Taking both findings into consideration, it seems that the major factor is the prevalence effect of the targets. In both experiments, high-prevalence items were found much more efficiently than low-prevalence targets. In both experimental and real-life situations, targets that have the most priority are more likely to be found. This might be one of the most important features of the incidental findings phenomena.

It is significant to note that the results in both experiments ultimately illustrated very similar tendencies, although target-distractor similarity was differed significantly. While in Experiment 2, targets belonged to a completely separate category in relation to distractors, items in Experiment 1 all constituted one category. It would seem, therefore, that the search in the second experiment would be far easier for the participants. However, this did not seem to play such a significant role. Firstly, the hybrid search paradigm in the first experiment is generally harder, since it involves searching from memory. Secondly, irrespective of task difficulty, the findings represented no significant decline in accuracy for finding the second target. Finally, despite the seeming target-distractor similarity distinction in the two experiments, the crucial point might be not categorical, but based on perceptual differences of the stimuli. Even though all items belonged to the same category of food in Experiment 1, they were very different perceptually. These characteristics could potentially be more important, since in real life the search task demands finding items with specific visual features. Such features may prevail over unclear categorical representations and, thus, guide the search for targets. However, there are data suggesting the overall categorical superiority in relation to perceptual phenomena in visual search (e.g., Biggs et. al., 2015). At the same time, this point needs further clarification, particularly with regards to incidental findings. Hence, a potential continuation of this study might be to vary the target-distractor similarity within one experiment in order to reveal the role of this particular factor.

Overall, the study revealed that incidental findings differ from SSM. This effect relates specifically to categorical visual search — a search defined by the category of objects. Additionally, prevalence of the targets plays an important role, since incidental findings relate to less common and less represented targets. These factors should be addressed by the optimal paradigm for studying incidental findings as a separate visual search phenomenon. Regarding the factor of target-distractor similarity, incidental findings seem to be perceptually different from the main targets of search. However, the specific role of perceptual differences should be clarified in further research.

## Conclusion

The purpose of this research was to study incidental findings in two separate experimental paradigms of visual search in order to reveal the primary factors specific to this phenomenon. The mixed hybrid search model and subsequent search misses (SSM) paradigm were used in two behavioral visual search experiments. The results revealed similar patterns in terms of participants’ accuracy and reaction time. The most significant factor for incidental findings was concluded to be the target prevalence effect. Targets that were more typically found seemed to create a certain bias towards similar items. However, rare targets that were more categorically distant from the initially identified target were more likely to be missed by the searcher. These results seem to be specific to incidental findings as opposed to SSM. Overall, the findings provide additional information about incidental findings as a separate visual search phenomenon.

## Limitations

Both experiments were conducted online due to the COVID-19 pandemic situation, which means that quite a few parameters of the experimental study could not be controlled. Those include the technical characteristics of the computers that varied from one participant to another, the conditions of noise and lighting, and others. Moreover, the display size was significantly reduced, since the majority of the participants had laptops with rather small screens. This implied a higher density of the stimuli on screen. In the case of visual search experiments, this might be an issue as it tends to make the task easier for the participants. However, since both experiments were very similar in technical aspects (e.g., stimuli size, grid parameters), it was possible to adequately compare the results. Moreover, since the critical parameter in both experiments was accuracy, rather than reaction time, and the results illustrated typical behavioral patterns for the described effects, the differences in technical parameters do not seem to have drastically influenced the data. As well as this, it has been argued that web-based experiments are appropriate for collecting such parameters as reaction time, even though they traditionally seem to be quite estimation-sensitive (Chetverikov & Upravitelev, 2015).
